# PERK eIF2 alpha kinase is required to regulate the viability of the exocrine pancreas in mice

**DOI:** 10.1186/1471-2121-8-38

**Published:** 2007-08-29

**Authors:** Kaori Iida, Yulin Li, Barbara C McGrath, Ami Frank, Douglas R Cavener

**Affiliations:** 1 Department of History of Science and Technology, Johns Hopkins University, Baltimore, MD 21218, USA; 2 Department of Biology, Penn State University, University Park, PA 16802, USA; 3 Bacterial Diseases of Livestock, Agricultural Research Service, United States Department of Agriculture, Ames, IA 50010, USA

## Abstract

**Background:**

Deficiency of the PERK eIF2α kinase in humans and mice results in postnatal exocrine pancreatic atrophy as well as severe growth and metabolic anomalies in other organs and tissues. To determine if the exocrine pancreatic atrophy is due to a cell-autonomous defect, the *Perk *gene was specifically ablated in acinar cells of the exocrine pancreas in mice.

**Results:**

We show that expression of PERK in the acinar cells is required to maintain their viability but is not required for normal protein synthesis and secretion. Exocrine pancreatic atrophy in PERK-deficient mice was previously attributed to uncontrolled ER-stress followed by apoptotic cell death based on studies in cultured fibroblasts. However, we have found no evidence for perturbations in the endoplasmic reticulum or ER-stress and show that acinar cells succumb to a non-apoptotic form of cell death, oncosis, which is associated with a pronounced inflammatory response and induction of the pancreatitis stress response genes. We also show that mice carrying a knockout mutation of PERK's downstream target, ATF4, exhibit pancreatic deficiency caused by developmental defects and that mice ablated for ATF4's transcriptional target CHOP have a normal exocrine pancreas.

**Conclusion:**

We conclude that PERK modulates secretory capacity of the exocrine pancreas by regulating cell viability of acinar cells.

## Background

Acinar cells of the exocrine pancreas synthesize and store copious quantities of the major digestive enzymes, poised to respond to episodic feeding events. Pro-enzymes (zymogens) are processed and folded in the rough endoplasmic reticulum and Golgi complex and are packaged into zymogen granules. Binding of a secretagogue such as cholecystokinin or acetylcholine to its receptors initiates Ca^2+ ^signaling and results in regulated exocytosis of zymogens into the duct. However, abnormal signaling caused by various factors including hyper-stimulation with high doses of secretagogues can activate zymogens prematurely in the pancreas, which results in autodigestion of the cells and acute pancreatitis.

Previously we [1], and others [2] have independently showed that the lack of PERK (EIF2AK3) in mice results in atrophy of the exocrine pancreas. This is consistent with earlier findings of pancreatic insufficiency in Wolcott-Rallison syndrome, a rare autosomal recessive disorder caused by a mutation in the human *Perk *gene [3,4]. PERK is an endoplasmic reticulum (ER)-resident kinase that phosphorylates eukaryotic translation initiation factor 2α (eIF2α) [5,6]. Hyper-activation of PERK in cultured cells by pharmacological ER-stress inducers results in global repression of protein synthesis and translational induction of ATF4 [7-9]. This, in turn, induces the transcription of GADD153/CHOP. Consequently, the observed atrophy of the exocrine pancreas, as well as the loss of the insulin-secreting β-cells of the endocrine pancreas, in the PERK-deficient animals was interpreted as a defect in the unfolded protein response (UPR) which resulted in uncontrolled ER-stress, followed by apoptotic cell death [2,10]. However, there is no direct evidence for uncontrolled ER-stress and apoptotic cell death in the exocrine pancreas of mice with knockout mutations of *Perk *(*PKO*), and recently the diabetes associated with PERK-deficiency in mice was shown to be caused by a proliferation and differentiation defect of the insulin secreting β-cells during fetal and neonatal development and not because of uncontrolled ER stress[11]. A confounding problem in assessing the defects in the exocrine pancreas of *PKO *mice is that a multitude of severe neonatal defects occur in other organs and tissues prior to the detection of overt anomalies in the exocrine pancreas[1,11]. These defects, which occur earlier in development, make it impossible to ascertain whether the anomalies occurring in the exocrine pancreas of *PKO *mice are cell-autonomous or secondary to dysfunctions in other organs.

To elucidate the mechanisms underlying the cell death and atrophy of the exocrine pancreas of the *PKO *mice, we have generated a pancreatic acinar cell-specific knockout mutation of the *Perk *gene (*exPKO*) in mice using the *Cre-loxP *system and have examined the critical physiological functions, the ER-stress pathway, and the mode of cell death in acinar cells.

## Results

### PERK is cell-autonomously required in the exocrine pancreas

We generated pancreatic acinar cell-specific *Perk *knockout mice by crossing a mouse carrying *Cre *recombinase driven by the *Elastase-1 *promoter ("*Ela-Cre*") [12] to a mouse carrying a *floxed Perk *gene [1]. The *Elastase-1 *promoter directs expression specifically in the rodent exocrine pancreas [12-14]. The tissue-specific knockout mice (*exPKO; Ela-Cre h+; Perk flox/flox*) showed a high CRE-mediated recombination rate (average 64.7% ± 0.06 [s.d.]) in the total pancreas at postnatal day 11 (P11). Since 7–8% of the total pancreas is comprised of other cell types we estimate that 70% of acinar cells contained the deletion of *Perk*. The fraction of phosphorylated eIF2α (to total eIF2α) in the *exPKO *pancreas is reduced to approximately 50% of wild-type at P42 but is somewhat higher than what is seen in *PKO *mice of the same age.

**Figure 1:**
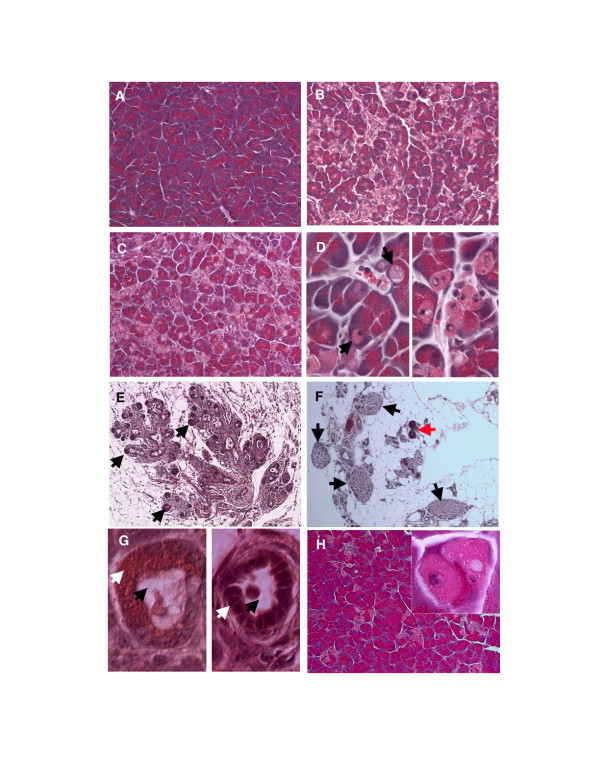
**Acinar cells are lost in both *PKO *and *exPKO *mice**. The PERK-deficient exocrine pancreas progressively loses acinar cells. Acinar cells are tightly packed in wild-type (A, P20). In contrast, *PKO *acinar cells have become degranulated giving a light pink appearance to the cytoplasm in the *PKO *pancreas (B, P20). The same phenotype is seen in acinar cell-specific PERK knockout (C, P19, *exPKO*). (D) Enlarged views of dying oncotic cells seen in (B) and (C). Some cells have lost nuclear staining (upper arrow) while others retain nuclear staining (lower arrow) (E) At P19, this particular *exPKO *mouse has already lost most of the exocrine pancreas although typically this degree of atrophy is not seen until 3–4 months of age. Arrows indicate examples of the smaller number of acini remaining. (F) In older mice (P162), acinar cells have been replaced by other cell types including adipocytes. Only a few dark pink acini are seen. Islets (arrows) still maintain an apparent normal structure. The animal also showed a normal glucose clearance rate. Red arrow indicates a few remaining acini. (G) In some cases, mutant acinar cells dedifferentiate into duct-like structures (P31) with abnormally large centroacinar ducts (black arrows). In the beginning of this process, duct cells still contain zymogen granules (right panel, white arrow). In some of these duct-like structures the presumptive acinar cells have completely lost zymogen granules (right panel, white arrow). (H) Conditional deletion of the *Perk *gene in 3-month-old *CreERT2; Perk flox/flox *mice also results in the appearance of oncotic cells. Two oncotic acinar cells are enlarged (inset) with lower left still exhibiting nuclear staining while the cell in the upper right shows a nuclear ghost. H&E staining. A-C, H, 200x; E, 100x; F, 80x; D, G, 600x.

**Figure 6:**
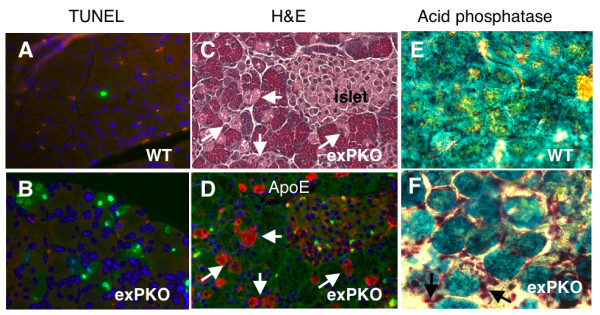
***exPKO *acinar cells die through oncosis. **(A, B) Acinar cells in the *exPKO *pancreas have numerous TUNEL-positive cells (A, P31 wild-type; B, P22 *exPKO*). Note that the TUNEL staining pattern strikingly differs between the two genotypes. TUNEL, green; DAPI, blue; background, red. (C, D) Adjacent serial sections (3μ) of the *exPKO *pancreas (P19). Pale pink (oncotic) acinar cells identified by H&E staining (C) correspond to cells positive for ApoE (D); see arrows as an examples of several cells. ApoE, red; DAPI, blue; background, green. (E, F) The KO pancreas has significant leukocyte infiltration (E, wild-type; F, *exPKO*, P74). Dark purple acid phosphatase staining indicates infiltrated leukocytes including macrophages (see arrows) in F but not E. The background cellular staining is methylene blue.

The *exPKO *and wild-type pups from crosses of *exPKO *(*Ela-Cre h+; Perk flox/flox*) × *Perk flox/flox *were born at the expected 1:1 Mendelian ratio. Unlike the *PKO *mice, the *exPKO *mice were fertile and indistinguishable in appearance from their wild-type littermates. Before P15, both *PKO *and *exPKO *mice showed normal exocrine pancreatic histology and expression levels of pancreatic amylase mRNA and protein (data not shown) were similar to that of wild-type animals. Proliferation of *exPKO *acinar cells was also normal as measured by BrdU incorporation (based on 4 mutant and 3 wild-type pancreata at P12-14; *P *= 0.63 [*n.s*.] by Student's *t*-test). However, at postnatal 3 weeks (typically at P19), the exocrine pancreas of both *PKO *and *exPKO *mice exhibited structural disorganization and cell death (Figure 1A to 1D). At this postnatal age 12% (± 0.09 [s.d.]) of the acinar cells were swollen and degranulated, lacking the intense staining of zymogen granules, and had abnormal nuclei with varied content density (Fig. 1D). The distribution of these cells was random across the entire pancreas. Initially defective cells were observed sporadically, and the number of acini containing those cells as well as the number of defective cells per acinus increased progressively across the entire exocrine pancreas without a particular pattern. Although the age of mutant phenotype onset varied, the cellular defect appeared by P25 with nearly complete penetrance. Because of extensive acinar cell death, the size of the pancreatic mass decreased with age. By 3–4 months, acinar cells were no longer the major cell type of the exocrine pancreas and had been replaced by fibroblasts, adipocytes, and macrophages/leukocytes (Figure 1E, F). Infiltration of macrophages and leukocytes was confirmed by staining for acid phosphatase, a marker for lysosomes enriched in macrophages (Figure 6E, F). A subset of severely affected mice had what appeared to be dedifferentiation of acini into duct-like structures in lieu of the cell death that was observed in most animals (Figure 1G).

The progression and magnitude of the cellular and molecular abnormalities of the exocrine pancreas were very similar in the *exPKO *and *PKO *mice, even though 30% of acinar cells in *exPKO *were estimated to be wild-type at P11. Two possible explanations for the equally severe atrophy in the *exPKO *mice are that the remaining wild-type cells (at P11) eventually undergo Cre-mediated deletion or that the dying PERK-deficient cells induce cell death of the remaining wild-type cells.

Unlike the PKO mice that have severely reduced β-cell mass and become diabetic by P22, the islets of Langerhans of the *exPKO *mice remained morphologically normal (Figure 1F) and exhibited normal distribution of α-glucagon and β-insulin cells (not shown). In addition the *exPKO *mice never became diabetic and exhibited normal glucose clearance rates at P22, P162 and P231 when subjected to a glucose tolerance test indicating that islet function was not perturbed (not shown). This was surprising because few acinar cells remained in these animals to provide the structure and typical cellular context for the islets (Figure 1F). These data suggest that the lack of PERK in acinar cells specifically affects the exocrine pancreas and does not contribute to the diabetes that develops in the *PKO *mice. In addition, expression of a *Perk *transgene in β-cells in the *PKO *background (β*-Perk; PKO*; see [15]) rescues islet mass and diabetes but not the exocrine atrophy. Furthermore, tissue-specific *Perk *ablation in the entire pancreas (*pcPKO*, using *Pdx1-Cre*) causes both diabetes and exocrine atrophy; by contrast, mice with gene deletion only in islets (*enPKO*, using *Ngn3-Cre*) exhibit diabetes without exocrine atrophy [11]. Thus PERK is required cell-autonomously in pancreatic acinar cells for their viability, and the multitude of other abnormalities of the *PKO *mice do not cause acinar cell death.

Acinar cell death in *exPKO *and *PKO *mice typically appeared around the neonatal-juvenile transition at postnatal 3 weeks. Numerous developmental and nutritional changes occur during this interval including a major transition in diet as pups are weaned from high-fat milk onto solid chow, which puts new demands on the exocrine pancreas. To test if the *Perk *mutant phenotype onset is affected by the dietary change we postponed weaning until after P21 when pups were past the age when the mutant phenotype is typically initiated. We found that delaying the dietary change that occurs at weaning does not prevent the progression of exocrine pancreatic atrophy in *exPKO *mice (not shown). To determine if the atrophy of the exocrine pancreas in PERK-deficient mice is developmentally regulated, we utilized a tamoxifen-inducible *Cre*-deletor strain targeted to the exocrine pancreas (*Elastase-CreERT2*) to conditionally mutate the *Perk *gene in adult mice. Multiple doses of tamoxifen were administered to 2–3 month-old mice bearing *floxed *alleles of *Perk *and the *Elastase-CreERT2 *transgene. Several acinar cells in the tamoxifen-treated mutant mice appeared degranulated, swollen and lacked normal nuclear content as observed in the *PKO *and *exPKO *mice (Figure 1H). As expected, the efficiency of CRE-mediated deletion of *Perk *was substantially lower than what was observed in the *exPKO *pancreas and consequently significantly fewer acinar cells succumbed to cell death. However, both gene deletion and the cell death were unique to mice carrying *CreERT2; Perk flox/flox *that were treated with tamoxifen and were absent in the tamoxifen-injected wild-type mice or non-injected controls. Therefore we conclude that PERK is required for maintaining the viability and function of the adult exocrine pancreas.

### *ExPKO *acinar cells show pancreatitis-like phenotypes

**Figure 2:**
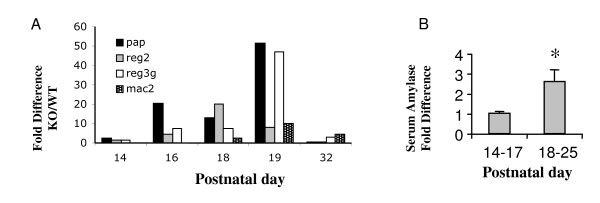
**The *exPKO *mice show pancreatitis-like phenotypes**. (A) Real-time RT-PCR quantification of pancreatitis markers, *Pap, Reg *and *Mac2*, in *exPKO *showing induction in acinar cells at P16-32. Ratio of KO to WT is shown. Each value is either a single individual or average of multiple individuals. Variation among individuals is large due to differences in when these genes are induced and repressed. All genes were normalized to the levels of tubulin mRNA amplified from the same sample. Wilcoxon paired sample sign test and Fisher sign test for the combined data set of these four genes showed highly significant bias (P < 0.0001, Wilcoxon test and P < 0.001, Signs test) toward increased expression of these genes during the P16–P32 postnatal period. (B) The serum amylase level of the *exPKO *mice is significantly increased in the age group after P18 (*, Student's *t*-test, *P *< .01). Samples were collected from 14 and 36 *exPKO *animals (P14-17 and P18-25, respectively). Amylase was assayed enzymatically as described in Methods. Average fold-differences are shown (wild-type = 1.0; Error bars = s.e.m).

To assess global changes in gene expression in the *exPKO *mice, we performed microarray analyses on RNA isolated from whole pancreata using Affymetrix mouse arrays (430A). The genes that showed the largest increase in the *exPKO *pancreas consistently at both P16 and P19 (as compared to the wild-type littermates) were the pancreatic stress response (PSR) genes including pancreatitis-associated protein (*Pap*) and regenerating islet-derived (*Reg*) *2 *and *3 *(see Additional file 1). The magnitude of the induction was large; for example, *Pap *was elevated by 26-fold, and *Reg3a *and *Reg3g *were also increased by more than 10-fold at P19. However by P32 these genes were no longer increased significantly in the *exPKO *pancreas, which is consistent with the accelerated loss of the acinar cells and the observation that these are acute-phase response genes [16]. Real-time quantitative RT-PCR analyses on animals of various ages confirmed that the mRNA levels of these genes were elevated as early as P16 in mutant pancreas (Figure 2A). The induction of the PSR genes was transient as shown by a rapid decline by P32. The transient induction of the PSR genes was associated with large variation among *exPKO *mice. We speculate that the large variation is exacerbated by the brevity of the period of induced gene expression. In contrast, few genes were consistently reduced in the mutant (see Additional file 2).

Induction of the PSR genes was observed 1–2 days prior to detection of any other exocrine pancreatic anomalies, including other hallmarks of acute pancreatitis such as elevation of serum amylase, cell death, and leukocyte infiltration. During acute pancreatitis amylase is released into circulation as acinar cells break down and leak cellular contents into their surroundings. Thus the presence of amylase in serum is indicative of acute pancreatitis. Serum amylase was elevated significantly in *exPKO *mice as early as P18 (typically at P19; Figure 2B). Elevation of serum amylase was tightly correlated with an increase in the number of acinar cells undergoing cell death. As cell death progressed, however, serum amylase returned to low but detectable levels (by P40; data now shown). This is most likely due to catastrophic loss of acinar cells, which also occurs in severe cases of chronic pancreatitis [17,18]. Serum amylase elevation and massive cell death were also accompanied by inflammatory responses. Gene profiling indicated the presence of an inflammatory response particularly at P19 and P32. Mutant samples showed a significant increase in genes predominately expressed in infiltrating macrophages and neutrophils such as *Mac-*2 (a macrophage marker), *Mmp12 *(macrophage elastase), lysosomal proteins (*Lip1 *and *Hexb*), and *Lcn2 *(neutrophil gelatinase-associated lipocalin precursor) (see Additional file 1; also see Figure 2A for *Mac-2*).

To summarize, the *exPKO *exocrine pancreas exhibits characteristics of acute pancreatitis and inflammatory responses. The induction of the acute PSR genes occurs first in the *exPKO *mice, followed by the appearance of a series of pathologies including cell death, serum amylase elevation, and inflammation. These events immediately preceded an observed increase in the number of cells succumbing to cell death at the end of the third postnatal week.

### *ExPKO *acinar cells show no evidence of ER-stress

**Figure 3:**
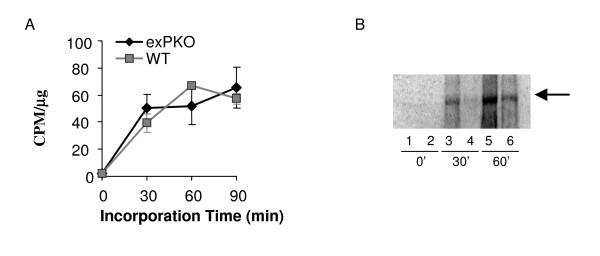
**Protein synthesis in *exPKO *acinar cells**. (A) Before the onset of massive cell death, protein synthesis is normal in the mutant pancreas. The graph shows the average of two independent experiments ([^35^S]Met/Cys incorporation) with a total of 4 replicates, using lobules isolated from P17 *exPKO *and wild-type littermates. TCA-precipitable radioactivity was normalized to the protein concentration of total lysate. (B) Protein synthesis in older *exPKO *mice is significantly reduced. Lobules were isolated from P63 *exPKO *(lanes 2, 4, 6) and wild-type (lanes 1, 3, 5) littermates and incubated for 0, 30, and 60 minutes with [^35^S]Met/Cys and 10 mg of total protein per lane were separated electrophoretically. The arrow indicates the position of amylase, which is the predominant zymogen in the exocrine pancreas and is readily detectable in whole radiolabeled protein. This gel was exposed for a short period and other proteins in this molecular weight range were not visible.

The widely accepted UPR model predicts that loss of PERK function results in uncontrolled translation leading to ER protein overload, up-regulation of the transcriptional arm of the ER-stress response and ultimately cell death [2]. To test if loss of PERK causes ER-stress *ex vivo *we assessed the characteristic outcomes of the ER-stress response in pancreatic lobules. Since PERK is predicted to directly regulate translation we first asked if protein synthesis was altered in the exocrine pancreas of PERK-deficient mice. Pulse-labeling experiments were conducted in lobules isolated from mice just prior to when overt cytological changes are first seen in mutants (P17) and protein synthesis rates were calculated. The protein synthesis rate for pancreatic lobules from P17 *exPKO *mice was indistinguishable from wild-type littermates, however, it was significantly diminished in older mutant animals (P63) (Figure 3A, B). The latter result is most likely due to the reduction in the number of acinar cells at this age.

**Figure 4:**
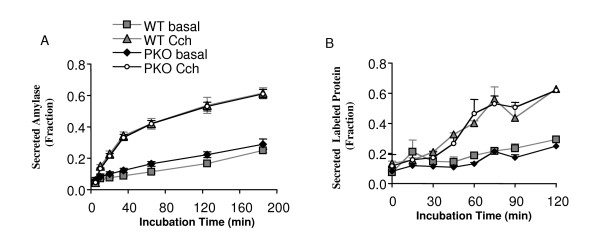
**Secretion is normal in *exPKO *acinar cells**. (A) Amylase secretion. The fraction of secreted amylase to total cellular amylase content was measured enzymatically for both basal and carbachol-stimulated pancreatic lobules. This is a representative result from 4 independent experiments. Lobules were isolated from P17 *β-Perk; Perk+/+ (WT) *and *β-Perk; Perk-/- (PKO)*. The β-Perk; Perk-/-*(PKO) *mice are rescued for *Perk *in endocrine β-cells with all other tissues remaining PERK-deficient, whereas the *β-Perk; Perk+/+ (WT) *control mice harbor the rescuing *Perk *transgene in an otherwise *WT *background. The *β-Perk; Perk-/- (PKO) *mice are analogous to the *exPKO *mice with respect to their exocrine pancreas. Each data point represents three replicate samples. (B) Pulse-chase analysis of protein secretion. The average of two independent experiments is shown. Lobules were isolated from *exPKO *(*PKO*) and wild-type (WT) littermates (P17-18). Lobules were labeled with [^35^S]Met/Cys as described in Figure 3 and then chased in "cold" media over 2 hours. The fraction of TCA-precipitable radioactivity in the media to total cellular TCA-precipitable counts was calculated for each time point for both "basal," (without a secretagogues) and secretagogues-stimulated conditions ("Cch"; with 0.5 μM carbachol).

To evaluate the function of the secretory pathway, secretagogue-induced exocytosis of amylase was examined in lobules isolated from P17 mice using 0.5 μM carbachol (acetylcholine analog). Basal secretion rates were not significantly different between the two genotypes and carbachol increased amylase secretion in both genotypes with similar magnitudes and timing (Figure 4A). These results indicate that the signaling cascade, from receptor recognition to extracellular release of zymogens, is normal in the PERK-deficient exocrine pancreas. To further test the functionality of the entire secretory pathway, pulse-chase analysis was performed using lobules isolated from P17-18 mice. Basal and stimulated secretion rates did not differ between genotypes, and the transit time of newly synthesized proteins was also similar (Figure 4B). This suggests that immediately prior to the onset of acinar cell death, both basal and secretagogue-stimulated secretory functions are normal in the *exPKO *mice.

**Figure 5:**
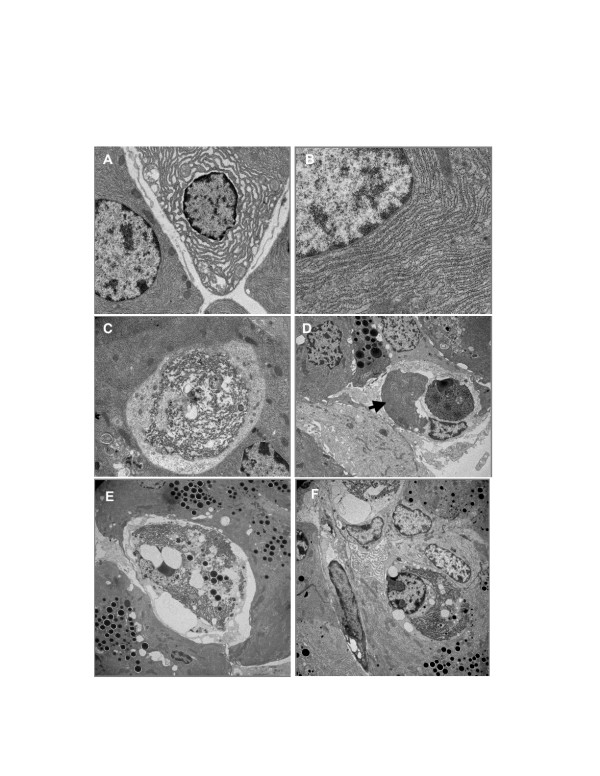
**The ER morphology is overall normal in *exPKO *acinar cells**. (A, B) Acinar cells of both wild-type and *exPKO *pancreata exhibit distended and prototypical ER at a similar frequency (A, P16 wild-type, 3000x; B, P16 *exPKO*, 7500x). (C) An *exPKO *acinar cell undergoing cell death (oncosis), P25 *exPKO*, 2500x. (D) The pancreas of the *exPKO *mouse (P25) contains infiltrating macrophages. The arrow points to the ER of an acinar cell engulfed by a macrophage. 1500x. Macrophages were not seen in the wild-type pancreas (data not shown). Also see Figure 6 for acid phosphatase staining. (E, F) Images of mutant cells undergoing cell death, 1200x. Note that these still retain the nucleus, which is vacuolated and does not contain chromatin condensation.

Previously it was reported that the ER was frequently distended in PERK-deficient acinar cells [1,2]. This observation has been interpreted as further evidence that mutant acinar cells experience unmitigated ER-stress and has also motivated subsequent studies that link ER distention to other cellular defects in PERK-deficient acinar cells [19]. However, a detailed histological examination of a greater number of pancreata revealed that acinar cells exhibiting lumenal distention were seen in wild-type and *exPKO *mice at equal frequency with no ascertainable differences in morphology (Figure 5A, B). In addition, the frequency of these cells in the *exPKO *pancreas did not increase as atrophy progressed (Figure 5C). Extensive monitoring of the ER ultrastructure during postnatal development failed to detect mutant acinar cells that appear to transition from a luminally distended state to cell death. Cells exhibiting distended ER by TEM averaged 10% of total immediately prior to (P16) and after (P25) phenotype onset. Originally, luminal ER distention in PERK-deficient acinar cells was claimed as prime evidence for severe abnormality in protein folding in the ER, leading to cell death in the mutant exocrine pancreas [1,2]. However, based on our observations, we conclude that this argument is incorrect.

Finally, in order to assess the level of ER-stress in acinar cells, the expression of the principal regulatory components and markers of ER-stress including *BiP*, *Chop*, and the spliced form of *Xbp1 *(*Xbp1-s*) were examined. The expression of these genes was not significantly altered in the total pancreas of *exPKO *mice. At P14-19, the average fold difference (± s.e.m., sample size) in mutants as compared to wild-type littermates was as follows: *BiP*, 1.21 (± 0.17, n = 7); *Chop*, 0.93 (± 0.22, n = 14); *Xbp1-s*, 1.15 (± 0.17, n = 11). This is consistent with microarray data that showed that expression of ER-resident and ER-stress-related genes was not significantly different between the mutant and wild-type pancreata at all ages examined (see Additional file 3), However, we did detect increases in mRNA levels of the pancreatic stress response (PSR) genes, which suggests that had ER-stress functions been similarly up-regulated we could have readily detected them as well.

### *ExPKO *acinar cells die through oncosis, not apoptosis

The previously reported high frequency of TUNEL-positive acinar cells found in *PKO *mice as early as P18 led to the conclusion that the mode of cell death was apoptosis [1,2]. However, it has become increasingly clear that the TUNEL assay cannot distinguish between apoptosis from other forms of cell death including oncosis [20-22]. The visual hallmarks of apoptosis are cellular shrinking and condensation of chromatin into apoptotic bodies, whereas oncotic cell death is characterized by cellular swelling, chromatin fragmentation without formation of apoptotic bodies, and loss of plasma membrane integrity. Necrosis has often been used synonymously with oncosis however it is now generally accepted that necrosis properly describes the morphological alterations that appear after cell death has occurred regardless of the mechanism [23]. TEM analyses on the mutant pancreata failed to detect apoptotic bodies despite the appearance of numerous TUNEL-positive cells by P25 (see Figure 6B). Instead, acinar cells in the P25 mutant pancreas undergo vacuolation followed by karyolysis (Figure 5C–F) which are characteristic of oncosis (reviewed in [24]). Closer observation revealed two distinct TUNEL staining patterns. In both wild-type and mutant acinar cells a very small fraction (< 0.1%) of acinar cells exhibited intensely stained nuclei and fragmented nuclei, which are characteristic of apoptotic nuclear bodies. The fraction of cells exhibiting such TUNEL positive nuclei did not differ between genotypes. In contrast in *exPKO *acinar cells, a high frequency of TUNEL positive cells were observed to have diffuse staining throughout both the nucleus and cytoplasm (Figure 6A, B), consistent with the loss of nuclear membrane integrity. Furthermore, microarray analysis did not detect increased expression of apoptosis-related genes before, during, or after the peak period of cell death in the exocrine pancreas (P16, 19, and 32; see Additional file 4). During the peak period, up to 6% of the mutant acinar cells were TUNEL-positive and at this frequency apoptosis-related genes should have been readily detected had they been up-regulated in the *exPKO *pancreas. Instead, all array data indicated a significant induction of inflammatory genes in the mutant pancreas but not apoptotic genes (see Additional file 1). Of the genes that showed 2-fold or higher elevation in the P19 mutant pancreas, 38% were related to inflammatory, immune, or acute PSR responses.

An additional distinguishing characteristic of oncotic cells is the loss of plasma membrane integrity that ultimately leads to inflammatory responses [24,25]. We developed a new technique to assess plasma membrane integrity in whole animal tissues based on the normal exclusion of the serum proteins apolipoprotein E, albumin, and IgG. In the normal exocrine pancreas, serum proteins are only found in the vasculature; however, in *exPKO *mice, serum proteins were detected in the degranulated acinar cells undergoing oncotic cell death (Figure 6C, D). Although serum proteins were abundantly detected in these cells, the mRNA levels were not elevated (not shown), thus eliminating the possibility of ectopic gene expression of these proteins. This observed loss of plasma membrane integrity as indicated by the passive uptake of serum proteins further substantiates our conclusion that the *exPKO *acinar cells succumb to oncosis. If apoptotic, the cells would have retained their plasma membrane integrity and been rapidly eliminated thus avoiding the induction of an acute inflammatory response. However, the degranulated acinar cells typically persisted for a few days before being phagocytosed, and the induction of an inflammatory response was clearly evident. Large numbers of infiltrating macrophages and neutrophils were detected in the *exPKO *pancreas by both acid phosphatase staining (Figure 6E, F), and by TEM (Figure 5D). Gene profiling also showed that transcripts for macrophage- and neutrophil-marker genes were elevated in the mutant pancreas (see Additional file 1). Phagocytosis of oncotic acinar cells by macrophages in mutant pancreata was also frequently observed by TEM (Figure 5D).

### ATF4-deficient mice exhibit defects in the development of the exocrine pancreas

**Figure 7:**
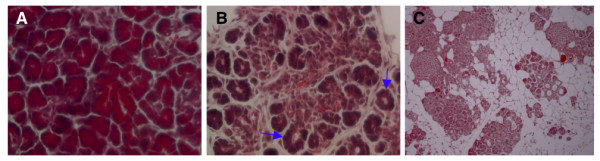
The *Atf4 KO *mice show pancreatic hypotrophy. As early as P4, *Atf4 KO *mice show fewer and smaller acinar cells in the exocrine pancreas (A, P4 wild-type; B, P4 *Atf4 KO*, 400x). The arrows point to tubular structures. By three months of age, the majority of the exocrine pancreas is replaced by adipocytes (C, *Atf4 KO*).

The translation of the ATF4 transcription factor is positively regulated by PERK activation in cultured fibroblasts subjected to ER-stress and results in the transcriptional upregulation of GADD153/CHOP. Consequently, ATF4 and CHOP have been considered to be prime targets of PERK-mediated regulation *in vivo *[7]. However, comprehensive evidence for this contention is lacking so we applied a genetic approach to ask if ATF4 and CHOP participate in the PERK's regulation of exocrine pancreas viability. First we examined *Atf4 *and *Chop KO *mice to determine if they displayed the same pancreatic defects as *Perk KO *mice. *Atf4 KO *mice exhibit postnatal growth retardation and are blind due to defective lens development [26,27]; however, pancreatic defects were not previously described. Surprisingly we found that the exocrine pancreata of neonatal (P4) *Atf4 KO *mice were severely underdeveloped and the number of acini and acinar cell size were greatly reduced (Figure 7). Typically, early neonatal mice have a large exocrine pancreatic mass with individual acini nearly contiguous, and as they mature, the acini become tightly packed. In contrast, the pancreatic acini of *Atf4 KO *mice were dispersed and often were not in close proximity to neighboring acini resulting in an expanded extracellular space. Moreover, *Atf4 KO *acinar cells appeared smaller with substantially less zymogen granule content, which correlated with an increase in the diameter in the centroacinar duct (Figure 7). In adults the centroacinar duct was greatly expanded resulting in highly tubular appearance of the exocrine pancreas. Adult *Atf4 KO *pancreata contained numerous adipocytes that appeared to have taken residence in the expansive extracellular space seen earlier in neonatal mice.

To determine if the reduction in acinar cell number in *Atf4 KO *mice was a consequence of cell death, TUNEL immunohistochemistry was performed. Unlike PERK-deficient mice, no increase in TUNEL-positive acinar cells was observed in *Atf4 KO *mice. As ATF4 is noted to regulate ER-stress response genes such as *BiP*, *Chop*, and *Xbp1-s *in fibroblasts, we measured the mRNA levels of these genes in *Atf4 KO *and wild-type pancreata to see if this regulation holds true for this tissue (Table 1). No significant differences were seen between *Atf4 *mutant mice and wild-type littermates for these ER-stress-related genes. To determine if *Atf4 KO *pancreata exhibit an inflammatory response similar to *exPKO *mice, the expression of *Reg2, Pap*, and *Mac-2 *mRNAs was quantified. Surprisingly, *Reg2*, which is potently up-regulated in *exPKO *mice, was substantially down-regulated in *Atf4 KO *mice. *Pap *was induced in the pancreas of *Atf4 KO *neonatal mice whereas *Mac-2*, a macrophage marker, was not induced.

**Table 1:** Gene expression in neonatal *Atf4 KO *mice as compared to wild-type littermates

	*BiP*	*Chop*	*Xbp1-s*	*Reg2*	*Pap*	*Mac-2*
Fold difference	0.84, *n.s*.	0.71, *n.s*.	0.83, *n.s*.	0.08*	4.93**	1.01, *n.s*.

*n.s.= *not significant

*P = 0.01

**P = 0.03

The ratios of KO to WT expression levels for P6-15 are shown. Statistically significant expression differences were determined by Student's *t*-test. The levels of all genes were normalized to *a-tubulin*, except *Xbp1*-s, which was normalized to *Xbp1-t*. For WT n = 6 and for KO n = 5.

CHOP upregulation is distal to both PERK and ATF4 activation and is generally considered to be pro-apoptotic, however, like caspase activation; it may also have a role in oncosis under certain circumstances [28]. It was therefore of interest to see if the exocrine pancreas of *Chop *knockout mice bore any resemblance to mice deleted for either PERK or ATF4. The pancreata of adult *Chop KO *mice were found to be completely normal (not shown). To test for the presence of genetic interactions between *Perk *and its putative downstream targets, we generated *Atf4+/-; Perk+/- *and *Chop+/-; Perk+/- *double-heterozygotes. These mice exhibited normal exocrine and endocrine pancreata (not shown) thus failing to show a genetic interaction for pancreatic development and/or viability.

Although we show for the first time that the exocrine pancreas of the *Atf4 KO *is greatly reduced, the underlying cause appears to be unrelated to the exocrine pancreas defect in *exPKO *mice. The deficiency in exocrine pancreas mass in *Atf4 KO *mice was observed early in postnatal development as a consequence of hypotrophy whereas the exocrine insufficiency of the *exPKO *mice was caused by death of mature acinar cells resulting in atrophy and a robust inflammatory response.

## Discussion

### PERK is required for the viability of pancreatic acinar cells

The generation of the exocrine pancreas-specific *Perk*-knockout mice enabled us to critically test whether exocrine pancreas defects seen in the global *PKO *mouse are the direct result of the loss of PERK expression in the acinar cells or are secondary to defects occurring earlier in other tissues or organs. We found that the *exPKO *mice had the same catastrophic loss of acinar cells as seen in global knockout mice, even though the deletion of *Perk *in the *exPKO *pancreas was incomplete (ca. 70%) at P11. We surmise that the nearly complete atrophy of the exocrine pancreas in *exPKO *is due to continued activity of the acinar-cell targeted Cre enzyme to delete the *Perk *gene in the remaining cells over the ensuing days or that the inflammatory response causes the death of the remaining wild-type cells as is seen when NFkappaB is overexpressed [29]. The remarkable consistency in mutant pancreatic phenotype among all of the lines that are ablated for *Perk *in the exocrine pancreas is a strong indication that expression of PERK in acinar cells is required for their viability. Although acinar cell death and atrophy were the predominant outcome of ablating PERK in the exocrine pancreas, a subset of severely affected animals harbored some acini that had apparently dedifferentiated into duct-like structures. This phenomenon is also seen in experimentally-induced pancreatitis [30] and in animals that have a genetic block in TGFβ signaling [31,32].

With the exception of the exocrine pancreatic deficiency, the *exPKO *mice did not display the other major defects associated with the Wolcott-Rallison syndrome in humans and the *PKO *mice. In some *exPKO *animals, the exocrine pancreas was replaced almost entirely by adipocytes, fibroblasts, and leukocytes, and the islets of Langerhans were no longer surrounded by acinar cells. These animals exhibited normal islet structure and glucose homeostasis. This demonstrates that the endocrine pancreas can survive and function without the exocrine pancreas being adjacent. It has been reported that chronic pancreatitis often results in diabetes, suggesting that dysfunction of the exocrine pancreas has deleterious effects on the endocrine pancreas (reviewed in [33]). In contrast, our findings suggest that the loss of the exocrine pancreas does not necessarily affect viability and function of the endocrine pancreas.

The onset of pancreatic atrophy in PERK-deficient mice occurs during the late neonatal stage of development and is first seen as sporadic degranulation of acinar cells followed by extensive cell death within a few days. Given the early postnatal onset of this atrophy, we considered whether these defects were dependent upon developmental age and nutrition. By delaying weaning, we showed that onset of exocrine pancreas atrophy in *exPKO *mice was not altered by changing the diet. In addition, we found that postponing the deletion of the *Perk *gene in the exocrine pancreas until the adult stage still lead to oncotic cell death, thus demonstrating that PERK is essential for the viability of the adult exocrine pancreas. In contrast, PERK is required for the fetal development and proliferation of the insulin-secreting β-cells of the endocrine pancreas, but is not required for β-cell viability in mature adults [11].

### The mode of cell death in PERK-deficient exocrine pancreas is oncosis

The acinar cells of the *exPKO *and *PKO *mice exhibit a dramatic increase in cell death. Initially we [1] and others [2] had proposed that the form of cell death was apoptosis but this was based entirely upon the detection of DNA fragmentation in the nuclei of acinar cells using TUNEL immunohistochemistry. It has been reported that TUNEL does not distinguish between apoptosis and other forms of cell death such as oncosis. Therefore, our conclusion that *exPKO *acinar cells succumb to oncosis rather than apoptosis is based primarily on morphological criteria [20-22]. Oncotic cells are characterized by cell swelling, vacuolization, chromatin fragmentation without formation of apoptotic bodies, and increased membrane permeability [24,25]. Fickert and colleagues [22] have recently shown that oncosis, not apoptosis, is the predominant form of cholestasis-induced cell death in the liver, and have provided a set of criteria for distinguishing between these forms of cell death in animal tissues that we have used herein.

Our conclusion that the mode of cell death in the PERK-deficient exocrine pancreas is oncosis derives from our key findings that include the appearance and progression of cell-swelling, karyolysis, and loss of plasma membrane integrity, as well as the induction of an inflammatory response and the absence of up-regulated apoptotic gene expression. Apoptotic cell death is often difficult to detect morphologically owing to the rapid cellular disintegration and immune clearance of the cells within a few hours. This rapid clearance and maintenance of plasma membrane integrity of apoptotic cells is thought to be a key factor limiting inflammation. In contrast, the oncotic acinar cells observed in the PERK-deficient exocrine pancreas seem to persist for a longer period as "ghost" cells that have lost plasma membrane integrity, and this may provide an opportunity for an inflammatory response to occur.

### The *exPKO *exocrine pancreas exhibits normal protein synthesis and secretion without any indication of ER-stress

The major function of PERK has been proposed to be the repression of global protein synthesis during ER stress and the activation of translation of specific genes that alleviate ER stress. Genetic ablation of PERK was therefore predicted to cause uncontrolled ER stress in normal cells resulting in heightened ER dysfunction and cell death. The gross pancreatic abnormalities seen in PERK-deficient mice, including low insulin-secreting beta cell mass and exocrine pancreatic atrophy, were initially assumed to confirm this hypothesis [1,2]. However the neonatal diabetes of PERK-deficient mice has been recently shown to be due insufficient proliferation and differentiation of the β-cells during fetal and neonatal development [34] and is not associated with either uncontrolled ER stress or cell death. In this study we also found that the exocrine pancreatic atrophy in PERK-deficient mice is inconsistent with the hypothesis that it is caused by uncontrolled or dysfunctional regulation of ER stress. First, protein synthesis and secretion are not significantly impaired in the mutant exocrine pancreas before or during the early phase of oncosis, although we assume that individual cells that have become degranulated and lost plasma membrane integrity are physiologically dysfunctional. Second, we did not observe an increase in expression of the major ER-stress genes. Instead, the genes induced in the mutant pancreas prior to overt cytological changes are the PSR genes. Third, knockout mutations in PERK's known downstream targets, *Atf4 *and *Chop*, do not result in cell death and atrophy of exocrine pancreas. Finally, the structure of the ER is normal in mutant pancreatic acinar cells prior to the onset of oncosis and does not exhibit distention as previously described [2]. Huang and coworkers [19] recently reported that PERK-deficient pancreatic acinar cells exhibit normal carbachol-stimulated ER Ca^2+ ^signaling as well as normal reloading of Ca^2+ ^following the response. The ability of exogenously supplied IP_3 _to induce ER Ca^2+ ^release was also normal. However, they did find that the periodicity of the Ca^2+ ^response was altered in Perk-deficient acinar cells and demonstrated that mutants have greatly reduced ER- plasma membrane interactions. They attribute these differences between mutant and wild-type to the previously reported distention and fragmentation of the ER in *PKO *acinar cells [2], a finding that our data now refutes. Yet overall the results of this study indicated that the receptor-mediated exocrine pathways are functional in Perk-mutant acinar cells, which is consistent with our finding that secretagogue-stimulated protein synthesis and secretion are normal in PERK-deficient acinar cells.

While we did not find evidence that ER stress was associated with pancreatitis in PERK-deficient mice, other studies have shown that induction of acute pancreatitis in wild-type rodents will lead to a classic ER stress response [35,36]. Caerulein hyperstimulation of acute pancreatitis in rats results in phosphorylation of eIF2α, and a reduction in total pancreatic protein synthesis [36], which is consistent with a potential role for PERK in mediating an ER stress response during an episode of acute pancreatitis. Another study using the arginine model of acute pancreatitis in rats found that all of the principle pathways of ER stress signaling including PERK activation and eIF2α phosphorylation were up-regulated and that characteristic ER stress-related apoptosis appeared shortly thereafter in the exocrine acini [35]. The differences between these acute models and what we observe in the *exPKO *pancreas may reflect overall differences in how pancreatitis is initiated and progresses. The hallmarks of pancreatic dysfunction in *Perk *mutant mice including the appearance of acinar cell degranulation, up-regulation of PSR genes and elevation of serum amylase take at least 2 weeks to appear whereas changes in pancreatic morphology, gene expression and the systemic manifestations of acute pancreatitis occur within hours of induction. It therefore seems reasonable to postulate that the signaling mechanisms responsible for pancreatitis in PERK-deficient mice differ from those that regulate the acute form of the disease in which PERK may participate. Common to all of these models of pancreatitis is the induction of inflammation that occurs in response to acinar cell death. In the animal models of acute pancreatitis inflammation has been commonly attributed to acinar cell necrosis; however based upon cytological descriptions these cells may be more properly considered oncotic [37].

We propose that PERK is required for the expression of anti-oncotic or pro-survival factors that are specific to the exocrine pancreas. PERK resides as a transmembrane protein kinase with its activation domain in the ER lumen and catalytic domain in the cytoplasm. PERK's activity is dynamically regulated by association/disassociation of the ER chaperone protein BiP/GRP78, whose activity is regulated by Ca^2+ ^as a cofactor and by ATP as a substrate. PERK is highly activated by pharmacological reagents that deplete the ER lumen of Ca^2+^, and we have shown that normal secretagogues known to mobilize ER Ca^2+ ^can also activate PERK under physiological conditions (B. McGrath and D. R. Cavener, unpublished data). These data suggest that PERK may be a sensor for Ca^2+ ^mobilization from the ER associated with secretory activity. We speculate that activation of PERK through normal secretory activity results in PERK-dependent regulation of anti-oncotic or pro-survival factors that maintain the viability of the acinar cells at adult stages. Necrosis is now generally regarded as the post-mortem state that results from uncontrolled cell death whether it arises from oncosis or as a secondary consequence of failure by the immune system to clear apoptotic cells in a timely fashion (secondary necrosis) [38]. However, recent reports have suggested that necrosis may also participate in regulated cell death associated with tissue renewal, embryogenesis, and immune response [25]. By analogy it is therefore, possible that a normal oncotic pathway exists in the exocrine pancreas and is misregulated in PERK-deficient mice.

## Conclusion

PERK is cell-autonomously required for the viability of the exocrine acinar cells but is not required for their normal protein synthesis and secretory functions. *Perk*-deficient acinar cells are lost by an oncotic mechanism of cell death and not by apoptosis as was originally reported [1,2]. Acinar death in *Perk *null mice is not associated with up-regulation of ER stress functions. Moreover mice carrying mutations in *Atf4 *or *Chop *which are known downstream targets of PERK signaling during ER stress do not recapitulate the *Perk *mutant exocrine phenotype.

## Methods

### Animals

All the procedures that involved animal subjects were approved by the Institutional Animal Care and Use Committee at the Pennsylvania State University to assure humane treatment of the animals. The knockout and floxed alleles of *Perk *in mice were previously described by us [1]. The floxed Perk allele contains two loxP sites located in intronic regions flanking three internal exons encoding segments of the lumenal and transmembrane domains. Cre-mediated deletion of these exons creates a frameshift resulting in a null phenotype. *Atf4 *and *Chop *knockout mice were obtained from Dr. Tim Townes, University of Alabama, and Dr. David Ron, New York University, respectively. For developmental stage-specific deletion of the *Perk *gene, tamoxifen (SIGMA) solution (20 mg/ml in corn oil) was injected (i.p.) into the *Elastase-CreERT2 *mice (2–3 months old) at 100 μl/animal [39]. To avoid tamoxifen-induced pancreatitis [40], the chemical was injected 12 times intermittently over one month. After 5 days from the last injection, the mice were sacrificed for further analyses.

For weaning-extension, two mating pairs per experiment (two females and one male) were set up in one large cage. The resulting litters were kept together with their dams until pups reached P10 when they were divided randomly into two cages: one with regular chow (as control) and the other without chow. Dams were switched between the two cages every 12 hours to let them access food. Water was freely accessible to mice in both cages. Conditions were maintained until the pups reached P25. Pups of the control group were weaned from the mother at P21, the normal age for weaning.

### Histological studies

Paraffin-embedded tissue was prepared as described previously [1]. Sections were made at 3–5 μ thickness (3 μ for serial sections). Fluorescent labeling was carried out using anti-ApoE (1:250; Santa Cruz) and an appropriate fluorescently-conjugated secondary antibody (1:500; Molecular Probes). Nuclei were labeled with DAPI (Molecular Probes). Cell proliferation was measured by in vivo labeling with 5-bromo-2'-deoxyuridine (BrdU; SIGMA). intraperitoneally injected at 50 mg/body weight (kg) Mice were sacrificed 24 hours post-injection and BrdU incorporation was detected using anti-BrdU (1:30; DAKO). DNA fragmentation was detected by TUNEL (TdT-mediated dUTP Nick-End Labeling; DeadEnd Fluorometric TUNEL system, Promega) following the manufacturer's protocol. A leukocyte marker, acid phosphatase, was detected using Naphthol AS-BI phosphoric acid solution (SIGMA). This assay was performed on frozen sections (10 μ), of tissues fixed in 4% phosphate-buffered paraformaldehyde for 2 hours at room temperature and then cryoprotected in 15% sucrose at 4°C overnight. Digital images were captured using a Nikon E1000 microscope and processed using the Image Pro (Phase 3 Imaging Systems) software. The background brightness and contrast were kept equivalent for all images that were directly compared so as not to distort any apparent differences. Samples for transmission electron microscopy (TEM) were prepared as described previously [1].

### Amylase assay

Serum samples were separated from cardiac blood and amylase activity was detected using Amylase Reagent (RAICHEM) according to the manufacturer's protocol. The fold difference was calculated by normalizing each mutant sample to the average of the wild-type samples within the same litter and the averages of the fold difference from multiple experiments is presented in Results.

### Lobule preparation

Pancreata were excised from mice that had been fasted overnight, and placed into a Petri dish containing KRB-HEPES buffer, 5 mg/ml bovine serum albumin (BSA; SIGMA), and 100 μg/ml soybean trypsin inhibitor (STI; SIGMA). The KRB-HEPES buffer contained: Krebs-Ringer bicarbonate (KRB) buffer mix (SIGMA), 0.9 mM CaCl_2_, 20 mM HEPES, and essential amino acids (GIBCO). The KRB-HEPES buffer with BSA and STI was injected to the tissue, and lobules were excised under a dissecting microscope [41].

### Protein synthesis and secretion experiments

Approximately 30 lobules per sample were deprived of methionine and cysteine (Met/Cys) for 10 minutes at 37°C in Met/Cys-free Dulbecco's Modified Eagle Medium, 20 mM HEPES, Aprotinin (25 μg/ml; SIGMA), STI (100 μg/ml), and BSA (5 mg/ml). The lobules were then labeled with [^35^S]Met/Cys (500 μCi/ml) at 37°C for 15 minutes. The reaction was stopped by the addition of concentrated non-radioactive Met/Cys solution (0.1 M each). After lobules were washed twice, chase buffer (KRB-HEPES buffer with BSA [5 mg/ml], STI [100 μg/ml], and Aprotinin [25 μg/ml]) and either carbachol (at 0.5 μM) or water (for control samples) was added. The samples were incubated in a shaking water bath (37°C; 120 rpm) and were oxygenated every 15–20 minutes. An aliquot of buffer was removed at different time points and later trichloroacetic acid (TCA)-precipitated using ProteoPrep Protein Precipitation Kit (SIGMA). At the end of the experiment, lobules were washed, homogenized, and total protein was TCA-precipitated. Precipitates were dissolved in 1% sodium dodecyl sulphate; 0.1 M Tris, pH 8.9, and the resident radioactivity were measured by scintillation counting.

The conditions used to measure incorporation of [^35^S]Met/Cys into total protein were the same as those used for the pulse-labeling experiment. All samples were TCA-precipitated, and the radioactivity was normalized to the protein concentration of the total lysate.

Measurements of secreted amylase were obtained from basal and carbachol-stimulated lobules subjected to non-radioactive "chase" conditions described above. In this case both buffer aliquots and lobule homogenate were assayed directly for amylase activity without prior TCA precipitation.

### RNA and DNA preparation

RNA samples were prepared using RNeasy Mini kit (QIAGEN). To prevent RNA degradation, the pancreas was excised and immediately placed in a mortar containing liquid nitrogen. The frozen tissue was crushed into small pieces, and homogenized in RLT buffer using QIAshredder columns (QIAGEN). The rest of the procedure followed the manufacturer's protocol. DNA samples for Real-time PCR were prepared using DNeasy Tissue kit (QIAGEN), following the manufacturer's protocol.

### Quantification of expression levels

Reverse transcription (RT) was carried out using M-MLV reverse transcriptase, buffer and Random Primers (Promega) according to the manufacturer's protocol. Real-time PCR of cDNA of all genes except *Perk *was carried out using qPCR Core Kit for SYBR Green I (Eurogentec). Reactions were amplified using the ABI Prism 7000 Sequence Detection System with the following program: 50°C (2 minutes), 95°C (10 minutes), 40 cycles of (95°C [15 seconds], 60°C [1 minute]). A linear amplification range was chosen for analyses using ABI Prism 7000 SDS Software (Applied Biosystems). The dissociation curves were monitored for the quality and specificity of amplification. Levels of *Xbp1-s *(spliced form) were normalized to *Xbp1-t *(total) levels. All other genes were normalized to the levels of tubulin. Fold-difference calculations were carried out within the same litter because variation among litters was too large to permit pooling across litters. The average of the fold-difference values across different litters from several replicate experiments is presented in Results. The primer sequences were as follows: *Bip*, 5'-ACCCTTACTCGGGCCAAATT-3', 5'-AGAGCGGAACAGGTCCATGT-3'; *Chop*, 5'-CCAACAGAGGTCACACGCAC-3', 5'-TGACTGGAATCTGGAGAGCGA-3'; *Mac-2*, 5'-GATGAAGAACCTCCGGGAAAT-3', 5'-GTTATGTCACCACTGATCCCCA-3'; *Pap*, 5'-CCAAAAGAGGCCTGGAGGAC-3', 5'-GCCTCAGCGCTATTGAGCAC-3'; *Reg2*, 5'-CTTCCCCTTGGCTGAAAAAGA-3', 5'-TCTGGGCAGTTGATTTTGGC-3'; *Reg3g*, 5'-ATGCTGCTCTCCTGCCTGAT-3', 5'-GGCAACTTCACCTTGCACCT-3'; *tubulin alpha*, 5'-CTTGGAACCCACGGTCATC-3', 5'-AAGAGCTGGCGGTAGGTGC-3'; *Xbp1-s*, 5'-GAGTCCGCAGCAGGTG-3', 5'-GTGTCAGAGTCCATGGGA-3'; *Xbp1-t*, 5'-CACCTTCTTGCCTGCTGGAC-3', 5'-GGGAGCCCTCATATCCACAGT-3'. Primer sequences for *Xbp1-s *were previously published [42].

Real-time PCR for the *Perk *gene was carried out as described previously [15]. The *Perk *"deleted region" primers amplify only the wild-type allele, whereas the *Perk *"total" primers amplify both wild-type and mutant alleles (*Perk *"deleted region", 5'-CAAACAAACCCAGCACCTTT-3', 5'-CATGGCCTGGCTTATTCTGT-3'; *Perk *"total", 5'-CATTCCTGGAACTGGGAGAA-3', 5'-CTCCATGGGACTCCTGGTTA-3'). The reactions were amplified using the GeneAmp 5700 DNA amplification system and analyzed with the GeneAmp 5700 SDS software (Applied Biosystems). To calculate the percentage of wild-type Perk allele remaining in the *exPKO *mice, the wild-type copy number estimated for the amplified "deleted region" was normalized to the copy number estimated using the "total" primers. Genomic DNA from wild-type, heterozygous and *PKO *were amplified simultaneously and used as standards to estimate the deletion frequency within the total *exPKO *pancreas. Theoretically, the percentage of wild-type allele left for the three standards should be approximately 100%, 50% and 0% respectively and this is what was observed.

### Microarray

All procedures followed the Affymetrix protocol. GeneChip Mouse Expression Array 430A was used for all samples. Affymetrix Microarray Suite 5.1 was used to calculate a difference in gene expression between *exPKO *and wild-type at P19 and P32. Detailed methods for sample preparation and determination of expression differences are included in Additional Data. Microarray results were deposited to the Gene Expression Omnibus database [43]Series #: GSE4422, 4423, 4424; Sample #: GSM99695, 99696, 99698, 99716, 99762, 99776, 99777, 99780.

## Authors' contributions

K. I. was responsible for generating and characterizing the tissue-specific and conditional *Perk *knockout animals, carried out protein synthesis and secretion studies, performed histological and electron microscopic examination of the exocrine pancreas and drafted the initial versions of this manuscript. Y.L. developed PCR-based methods for determining the extent of *Perk *deletion in tissue-specific and conditionally mutant animals and assisted with surgical and injection procedures. B.C.M. assisted with radioisotope labeling experiments, and participated in the drafting and revision of this manuscript. A.F. participated in the extensive histological examination of the exocrine pancreas. D.R.C conceived of the study and supervised its execution as well as drafting and editing of this manuscript. All authors read and approved the final manuscript.

## Supplementary Material

Additional file 1 Summary of microarray data for genes that show increased expression in mutant pancreata. A table listing genes that were shown to be up-regulated in the pancreata of *Perk-/- *mice compared to wild-type at P16, P19 and P32. Click here for file

Additional file 2 Summary of microarray data for genes that show decreased expression levels. A table listing genes that were shown to be down-regulated in the pancreata of *Perk-/- *mice compared to wild-type at P16, P19 and P32. Click here for file

Additional file 3 Results of microarray analyses for major ER-resident and ER-stress related genes. A table that compares known ER-resident or ER-stress related genes for *Perk-/- *and wild-type mice at P16. P19 and P32. Click here for file

Additional file 4 Results of microarray analyses for major apoptosis-related genes. A table that compares known apoptosis-related genes for *Perk-/- *and wild-type mice at P16. P19 and P32. Click here for file
