# Supplementary material for: PERK eIF2 alpha kinase is required to regulate the viability of the exocrine pancreas in mice
Source: BMC Cell Biol. 2007 Aug 29;8:38. doi: 10.1186/1471-2121-8-38 (PMC2072952; doi:10.1186/1471-2121-8-38)
Supplement: Additional file 1 — Summary of microarray data for genes that show increased expression in mutant pancreata. A table listing genes that were shown to be up-regulated in the pancreata of Perk-/- mice compared to wild-type at P16, P19 and P32. [file 1471-2121-8-38-S1.pdf]

Gene symbol	Gene title	P16[a]	P19[a]	P32[a]
Stress/injury				
Pap	pancreatitis-associated protein	11.2	26.0	ns
Pap	pancreatitis-associated protein	7.2	13.0	ns
Reg3g	regenerating islet-derived 3 gamma	7.6	18.4	3.2
Reg3a	regenerating islet-derived 3 alpha	3.2	11.3	ns
Reg2	regenerating islet-derived 2	4.1	5.3	0.5
Hspa1a / Hsp70-3	heat shock protein 1A	10.5	-	-
Hspa1b / Hsp70-1	heat shock protein 1B	12.6	2.8	ns
Hspa1b / Hsp70-1	heat shock protein 1B	8.5	2.6	2.3
Hspa1b / Hsp70-1	heat shock protein 1B	6.5	2.5	ns
Hsp105	heat shock protein 105	ns	7.5	ns
1810009J06Rik	trypsinogen	ns	4.3	3.2
Ctrc	chymotrypsin C (caldecrin)	4.9	ns	0.2
Cox7a1	cytochrome c oxidase, subunit VIIa 1	-	2.6	5.3
Cp	ceruloplasmin	ns	ns	4.9
Inflammation/immune				
Mac-2 / Lgals3	lectin, galactose binding, soluble 3	ns	5.7	4.6
Mmp12	matrix metalloproteinase 12	-	14.9	55.7
Scya6 / Ccl6	chemokine (C-C motif) ligand 6	2.0	4.9	3.5
Lcn2	lipocalin 2	2.8	ns	4.3
Lip1	lysosomal acid lipase 1	ns	4.9	ns
Hexb	hexosaminidase B (lysosome)	ns	3.0	5.3
Serpine2	serine (or cysteine) proteinase inhibitor, E2	ns	2.0	4.3
Socs3	suppressor of cytokine signaling 3	ns	ns	6.5
Ambp	alpha 1 microglobulin/bikunin	ns	ns	4.3
Tlr3	toll-like receptor 3	ns	9.8	-
Pld1	phospholipase D1	ns	-	7.5
Matrix/cytoskeleton				
Bgn	biglycan	-	7.0	7.5
Vwf	Von Willebrand factor homolog	-	12.1	-
Gpnmb	glycoprotein (transmembrane) nmb	-	-	7.0
Cldn2	claudin 2	-	-	4.6
Cnn3	calponin 3, acidic	5.8	3.2	ns
Tagln	transgelin	-	4.3	-
Pfn2	profilin 2	ns	ns	4.3
Growth-related				
Tgfbi	transforming growth factor, beta induced	4.1	-	-
Acvrinp1	activin receptor interacting protein 1	6.2	-	ns
Pdgfra	platelet derived growth factor receptor alpha	ns	4.9	ns
Others				
Galgt1	beta-1,4 N-acetylgalactosaminyltransferase	-	ns	11.3
Gif	gastric intrinsic factor	-	-	9.2
Ms4a7	membrane-spanning 4-domains, A7	-	-	8.6

Ms4a11	membrane-spanning 4-domains, A11	ns	8.0	2.0
Thoc4	THO complex 4	ns	7.5	ns
Trim30	tripartite motif protein 30	ns	4.0	ns
Tm7sf1	transmembrane 7 superfamily member 1	ns	9.2	7.5
Tm7sf1	transmembrane 7 superfamily member 1	-	ns	7.5
Tmem45a	transmembrane protein 45a	-	ns	7.5
Tspan1	tetraspan 1	-	-	26.0
1700029I01Rik	RIKEN cDNA 1700029I01 gene	ns	ns	5.7
1110021E09Rik	weakly similar to splicing factor, arginineserine-rich 4	ns	ns	6.1
-	weakly similar to env polyprotein, retrovirus-related	-	ns	8.0
-	expressed sequence AI594671	5.7	-	-

Table S1. Summary of microarray data for genes that showed increased expression levels in the mutant pancreata. Fold change in the mutant expression levels as compared to the wild-type levels. "-", the expression was not detectable at a significant level; "ns," the expression was detectable but was less than 2-fold change between mutant and wild-type samples significant level (i.e., less than 2-fold change). Repeated appearance of the same gene in the tables indicates multiple detections by different Affymetrix probes for the same gene. For P16 samples, RNA from a single pancreas was used per array, and each genotype had two biological replicates. For data analysis, signals of the replicates were averaged for each probe within a genotype for calculation of a ratio (i.e., mutant average signal / wild-type average signal). Genes identified by the software as "Present" in at least one array out of a total of 4 arrays were selected for further analyses, and those with ratios ≤ 0.5 or ≥ 2.0 were considered as differentially expressed genes at a significant level. For P19 and P32 experiments, cDNA sample was similarly generated from total pancreatic RNA (10 μg) from single animals and then, equivalent amounts of two samples of the same genotype were pooled prior to cRNA and hybridization to a single array. Genes identified as "Present" in at least one of the two arrays (mutant and wild-type) were selected for further analyses. The genes that showed an increase difference of ≥ 2.0 fold in the magnitude (i.e., Signal Log Ratio ≥ 1.0 or ≤ -1.0) were considered as differentially expressed genes at a significant level. Repeat appearance of the same gene in the tables indicates multiple detections by different Affymetrix probes for the same gene.
