# Supplementary material for: PERK eIF2 alpha kinase is required to regulate the viability of the exocrine pancreas in mice
Source: BMC Cell Biol. 2007 Aug 29;8:38. doi: 10.1186/1471-2121-8-38 (PMC2072952; doi:10.1186/1471-2121-8-38)
Supplement: Additional file 2 — Summary of microarray data for genes that show decreased expression levels. A table listing genes that were shown to be down-regulated in the pancreata of Perk-/- mice compared to wild-type at P16, P19 and P32. [file 1471-2121-8-38-S2.pdf]

Gene symbol	Gene title	P16	P19	P32
Star	steroidogenic acute regulatory protein	-	0.2	-
Cyp39a1	cytochrome P450, family 39, subfamily a, polypeptide 1	ns	ns	0.2
Mat1a	methionine adenosyltransferase I, alpha	ns	ns	0.2
Alb1	albumin 1	0.2	-	-
---	RIKEN cDNA 6330416G13 gene	ns	-	0.3
Hamp2	hepcidin antimicrobial peptide 2	ns	ns	0.1
Fabp1	fatty acid binding protein 1, liver	0.0	-	-
Upp2	uridine phosphorylase 2	0.2	ns	0.4
Tdh	L-threonine dehydrogenase	ns	0.2	-
1810008K16Rik	ER resident transmembrane homologous to yeast ERP-6, emp24/gp25L/p24	ns	0.2	0.1
---	RIKEN cDNA 2310014L17 gene	ns	0.3	ns
---	RIKEN cDNA A030012M09 gene	ns	ns	0.3
Eif2ak3	eukaryotic translation initiation factor 2 alpha kinase 3	0.4	ns	0.2
Serpina1a	serine (or cysteine) proteinase inhibitor, clade A, member 1a	0.0	-	-
Ahsg	alpha-2-HS-glycoprotein	0.1	-	-
Tde1	tumor differentially expressed 1	0.2	ns	ns
Vegfb	vascular endothelial growth factor B	ns	-	0.2
Camk2b	Calcium/calmodulin-dependent protein kinase II, beta	ns	0.4	0.0
Myh6	myosin, heavy polypeptide 6, cardiac muscle, alpha	-	-	0.1
Vtn	vitronectin	ns	ns	0.2
Vtn	vitronectin	ns	ns	0.2
Gngt1	guanine nucleotide binding protein (G protein), gamma transducing activity polypeptide 1	ns	ns	0.3
Pou2f1	POU domain, class 2, transcription factor 1	ns	ns	0.3
Eomes	eomesodermin homolog (Xenopus laevis)	-	-	0.1
---	RIKEN cDNA 2210418O10 gene	ns	0.5	0.2
BC013476	similar to cytochrome P450, 4a10	ns	-	0.2
---	similar to ubiquinol-cytochrome c reductase complex (7.2 kD)	ns	0.1	0.3
Pnrc1	proline-rich nuclear receptor coactivator 1	0.2	-	ns
Dtl	denticleless homolog (Drosophila), 2810047L02Rik	0.2	-	-

Table S2. Summary of microarray data for genes with decreased expression levels. Numbers are fold changes in gene expression levels in the mutant pancreata as compared to wild-type controls. Genes that showed reduced expression levels by 4 fold or higher in at least one age group are shown. "-," expression was not detectable at a significant level; "ns," expression was detectable but was not identified as significantly different between mutant and wild-type samples (i.e., less than 2-fold change). For P16 samples, RNA from a single pancreas was used per array, and each genotype had two biological replicates. For data analysis, signals of the replicates were averaged for each probe within a genotype for calculation

of a ratio (i.e., mutant average signal / wild-type average signal). Genes identified by the software as "Present" in at least one array out of a total of 4 arrays were selected for further analyses, and those with ratios ≤ 0.5 or ≥ 2.0 were considered as differentially expressed genes at a significant level. For P19 and P32 experiments, cDNA sample was similarly generated from total pancreatic RNA (10 μg) from single animals and then, equivalent amounts of two samples of the same genotype were pooled prior to cRNA and hybridization to a single array. Genes identified as "Present" in at least one of the two arrays (mutant and wild-type) were selected for further analyses. The genes that showed a decrease difference of ≥ 2.0 fold in the magnitude (i.e., Signal Log Ratio ≥ 1.0 or ≤ -1.0) were considered as differentially expressed genes at a significant level. Repeat appearance of the same gene in the tables indicates multiple detections by different Affymetrix probes for the same gene.
