# Supplementary material for: PERK eIF2 alpha kinase is required to regulate the viability of the exocrine pancreas in mice
Source: BMC Cell Biol. 2007 Aug 29;8:38. doi: 10.1186/1471-2121-8-38 (PMC2072952; doi:10.1186/1471-2121-8-38)
Supplement: Additional file 3 — Results of microarray analyses for major ER-resident and ER-stress related genes. A table that compares known ER-resident or ER-stress related genes for Perk-/- and wild-type mice at P16. P19 and P32. [file 1471-2121-8-38-S3.pdf]

Table S3. Results of microarray analyses for major ER-resident and ER stress-related genes.
Signal intensity, Present Call by Affymetrix software (P, present; A, absent, M, marginal), fold change, and indication of significance (*) are shown.
When a gene is either "A" or "M" in both genotypes, the fold change column has no value ("-").
Significance was indicated for the genes that had detectable expression levels and showed at least 2-fold difference between mutant and wild-type samples.
(Also see Materials and Methods for details.)

	P16						P19							P32							
Gene Name	E+_ave signal	P/A	E-_ave signal	P/A	Fold	sig*	E+_Signal	E+_	E-_Signal	E-_	Fold	Change	sig*	E+_Signal	E +_	E-_Signal	E -_	Fold	Change	sig*	Probe Set ID
Atf3	98.1	P	76.2	A	1.29		112.1	A	65.8	A	-	-		86.2	A	122.8	A	-	-		1449363_at
Atf6	279.2	P	217.7	P	1.28		230.9	P	224.5	P	1.00	NC		118.5	P	289.6	P	0.47	D	*	1435444_at
BiP	6039.0	P	6017.9	P	1.00		6863.9	P	4354.4	P	1.52	I		2099.7	P	4671.6	P	0.62	NC		1416064_a_at
BiP	3282.3	P	2897.4	P	1.13		2843.7	P	1450.4	P	2.00	I	*	759.7	P	1146.9	P	0.57	NC		1427464_s_at
Chop	111.5	P	86.2	P	1.29		63.7	A	67.2	A	-	-		121.2	P	148.4	M	0.81	NC		1417516_at
EDEM	832.0	A	715.4	A	-		570.2	A	571.7	A	-	-		253	A	630.6	A	-	-		1451218_at
EDEM	1607.7	P	1641.8	P	0.98		1824.2	P	1812.7	P	1.00	NC		1060.2	P	1768.5	P	0.57	NC		1424065_at
ERdj4	1515.7	P	1319.5	P	1.15		1075.6	P	1254.7	P	0.87	NC		441.5	P	744.6	P	0.66	NC		1417191_at
ERdj5	94.4	A	77.0	A	-		97.5	A	89.4	A	-	-		79	A	85.7	A	-	-		1452230_at
ERdj5	170.3	P	164.8	P	1.03		142.8	P	208.7	P	1.00	NC		99.4	P	124.6	A	0.87	NC		1426904_s_at
ERdj5	325.4	P	317.3	P	1.03		361.3	P	276.9	P	1.23	NC		251.7	P	281.4	P	0.93	NC		1426905_a_at
Ero1a	210.0	P	195.4	P	1.07		204.3	P	200.8	P	1.07	NC		127.6	P	224.8	P	0.54	NC		1419029_at
Ero1a	127.8	P	133.8	P	0.96		152.2	P	174.2	P	0.81	NC		58.3	P	120.1	P	0.54	D		1419030_at
Ero1a	401.5	P	467.6	P	0.86		432.7	P	464.4	P	0.93	NC		252.2	P	335	P	0.66	NC		1449324_at
Ero1b	94.3	A	94.5	A	-		130.4	A	96.7	A	-	-		82.5	M	127.6	P	0.62	NC		1449948_at
Ero1b	644.8	P	655.7	P	0.98		532.4	P	909	P	0.71	D		446	P	1023.9	P	0.38	D	*	1425705_a_at
Ero1b	2348.4	P	2686.5	P	0.87		2437.4	P	3058.5	P	0.87	NC		1554.1	P	3736.7	P	0.44	D	*	1434714_at
ERp29	642.2	P	618.1	P	1.04		648.8	P	692.5	P	0.93	NC		350.6	P	443.2	P	0.62	NC		1453634_a_at
ERp44	202.6	P	204.6	P	0.99		122.8	P	230.8	P	0.71	NC		136.2	A	175.8	A	-	-		1423246_at
ERp44	618.8	P	466.5	P	1.33		441.9	P	358.2	P	1.07	NC		326	P	339.6	P	0.87	NC		1423247_at
ERp72	308.6	P	235.6	P	1.31		173.7	P	71.5	A	1.74	I		142.9	P	146.4	M	1.00	I		1416497_at
Grp58	2085.2	P	1853.4	P	1.13		1312.6	P	664.6	P	2.14	I	*	508.8	P	944.7	P	0.76	NC		1423423_at
Grp94	1967.1	P	2091.2	P	0.94		2063.3	P	1477.3	P	1.52	I		702.9	P	1126.4	P	0.66	NC		1415889_a_at
Grp94	1381.1	P	1428.0	P	0.97		1153.3	P	908.5	P	1.41	I		268.1	P	532.9	P	0.57	NC		1438040_a_at
HEDJ	1254.3	P	1170.6	P	1.07		749.9	P	731.3	P	1.15	NC		372	P	636.7	P	0.62	NC		1423151_at
Herp	4606.8	P	4347.8	P	1.06		2894.4	P	3493.6	P	0.93	NC		2013.7	P	4892.8	P	0.47	MD		1435626_a_at
Herp	3315.6	P	3724.5	P	0.89		2221.8	P	2356.9	P	0.93	NC		1310.7	P	3102.3	P	0.47	NC		1448185_at
Ire1a	17.3	A	38.8	A	-		53.8	A	19.9	A	-	-		9.8	A	23.1	A	-	-		1421240_at
Ire1a	3.0	A	3.5	A	-		4.1	A	3.8	A	-	-		1.6	A	8	A	-	-		1431886_at
Ire1a	29.8	A	57.2	P	0.52		63.8	A	23.3	A	-	-		39.1	P	42.7	A	0.66	NC		1450176_at
Ire1b	4.1	A	6.9	A	-		8.7	A	41.4	A	-	-		5.8	A	4.3	A	-	-		1450139_at
p58IPK	987.9	P	911.0	P	1.08		1156	P	736.7	A	1.23	I		279.7	A	472	A	-	-		1419163_s_at
p58IPK	458.8	P	465.3	P	0.99		397.8	P	243.4	P	1.52	I		193.1	P	323.1	P	0.62	NC		1419162_s_at
p58IPK	1510.5	P	1221.5	P	1.24		1163.9	P	949.5	P	1.23	I		423.9	P	885.3	P	0.57	NC		1433887_at
p58IPK	1478.2	P	1242.7	P	1.19		1200.5	P	1062.9	P	1.15	NC		805.6	P	1216	P	0.62	NC		1449372_at
p58IPK	151.7	P	219.0	P	0.69		133.9	P	97.3	P	1.15	NC		40	P	85.9	P	0.54	NC		1449373_at
PDI	8537.1	P	9189.5	P	0.93		8808.6	P	10389.2	P	0.93	NC		5974.2	P	11407.6	P	0.54	NC		1437465_a_at
PDI-P5	68.7	P	63.2	A	1.09		70.8	A	77	A	-	-		39.1	A	75.4	A	-	-		1430983_at
PDI-P5	2440.1	P	2325.4	P	1.05		2033	P	1837.1	P	1.15	NC		1103.5	P	1204.1	P	0.87	NC		1423648_at
Ramp4	2438.6	P	2312.9	P	1.05		2439.5	P	2657	P	0.93	NC		1208.8	P	2237.4	P	0.50	NC		1415827_a_at
Ramp4	3546.2	P	3484.8	P	1.02		2828.3	P	3834	P	0.76	NC		1880.1	P	4077.8	P	0.47	NC		1415828_a_at
Sec61a1	1380.3	P	1243.1	P	1.11		1158.7	P	1242.4	P	1.07	NC		862.6	P	1383.4	P	0.62	NC		1416189_a_at
Sec61a1	625.0	P	599.0	P	1.04		418.3	P	546.1	P	0.71	D		250.5	P	539.9	P	0.47	D	*	1416190_a_at
Sec61a1	2146.2	P	2202.3	P	0.97		1373.2	P	1726.1	P	0.81	NC		701.7	P	1597.8	P	0.47	D	*	1416191_at
Sec61a1	1142.8	P	1035.5	P	1.10		784.4	P	784.9	P	0.93	NC		533.6	P	1090.2	P	0.47	NC		1434986_a_at
Sec61a1	1668.7	P	1447.0	P	1.15		1373.1	P	1385.9	P	1.07	NC		714.5	P	1067.6	P	0.66	NC		1448242_at
Sec61a1	334.2	P	357.5	P	0.93		397.6	P	413	P	1.00	NC		164.6	P	243.1	P	0.62	NC		1455987_at

Sec61a2	52.6	A	61.2	A	-	69.5	A	56.3	A	-	-	35.3	A	72.7	A	-	-	1449944_a_at
Sec61a2	47.3	P	58.0	A	0.82	45.5	A	66.4	A	-	-	35.1	M	69	A	-	-	1420644_a_at
Sil1	405.8	P	395.2	P	1.03	509.3	P	426.6	P	1.00	NC	309.1	P	455.2	P	0.62	NC	1417354_at
Xbp1	138.8	P	197.3	P	0.70	232	P	208.9	A	1.32	NC	163.9	P	208.3	P	0.81	NC	1420012_at
Xbp1	1052.6	P	974.5	P	1.08	1258.7	P	1016.2	P	1.23	I	976.4	P	1456	P	0.66	NC	1420011_s_at
Xbp1	4338.9	P	3879.3	P	1.12	5533.9	P	4178.6	P	1.32	I	4827.4	P	7590.7	P	0.62	NC	1420886_a_at
Xbp1	3617.4	P	3471.0	P	1.04	4928.5	P	3638.8	P	1.32	I	4141.7	P	5769.2	P	0.71	NC	1437223_s_at
