# Supplementary material for: PERK eIF2 alpha kinase is required to regulate the viability of the exocrine pancreas in mice
Source: BMC Cell Biol. 2007 Aug 29;8:38. doi: 10.1186/1471-2121-8-38 (PMC2072952; doi:10.1186/1471-2121-8-38)
Supplement: Additional file 4 — Results of microarray analyses for major apoptosis-related genes. A table that compares known apoptosis-related genes for Perk-/- and wild-type mice at P16. P19 and P32. [file 1471-2121-8-38-S4.pdf]

Table S4. Results of microarray analyses for major apoptosis-related genes.
Signal intensity, Present Call by Affymetrix software (P, present; A, absent, M, marginal), fold change, and indication of significance (*) are shown.
When a gene is either "A" or "M" in both genotypes, the fold change column has no value ("-").
Significance was indicated for the genes that had detectable expression levels and showed at least 2-fold difference between mutant and wild-type samples.
(Also see Materials and Methods for details.)

	**P16**						**P19**							**P32**							
Gene Symbol (	E+_ave signal	P/A a	E-_ave signal	P/A a	Fold	sig*	E+_Signal	E+_	E-_Signal	E-_	Fold	Change	sig*	E+_Signal	E +_	E-_Signal	E -_	Fold	Change	sig*	Probe Set ID
Apaf1	45.1	A	48.0	A	-		42.8	A	17.4	A	-	-		21.6	A	23.8	A	-	-		1450223_at
Apaf1	76.4	P	85.0	P	0.90		70.2	P	87.9	P	0.66	NC		63	P	58.1	P	0.81	NC		1452870_at
Bad	141.6	A	77.4	A	-		61.1	A	104.7	A	-	-		48.6	A	37.7	A	-	-		1416582_a_at
Bad	90.9	A	69.8	A	-		54.7	A	58.3	A	-	-		94	A	114.4	A	-	-		1416583_at
Bak1	23.8	A	28.1	A	-		11	A	10.6	A	-	-		22.2	A	10.4	A	-	-		1425716_s_at
Bak1	47.1	P	36.7	A	1.29		39.8	A	17.2	A	-	-		28	A	5.8	A	-	-		1418991_at
Bax	172.2	P	172.2	P	1.00		124.1	P	155.8	P	1.00	NC		145.5	P	194.3	P	0.76	NC		1416837_at
Bcl2	107.7	A	130.3	A	-		195.4	A	169.3	A	-	-		87	A	193	A	-	-		1422938_at
Bcl2	24.7	A	47.4	A	-		65.8	A	40.2	A	-	-		40.1	A	64.8	A	-	-		1427818_at
Bcl2	39.6	A	41.6	A	-		45.7	A	24.5	A	-	-		28.9	A	29.8	A	-	-		1427819_at
Bcl2a1a /// Bcl	6.8	A	20.6	A	-		25.6	A	29.5	A	-	-		11.4	A	2.5	A	-	-		1450812_at
Bcl2a1a /// Bcl	52.9	A	39.8	A	-		93.4	A	67.5	A	-	-		72.5	A	73.2	A	-	-		1419004_s_at
Bcl2a1a /// Bcl	18.3	A	16.6	A	-		6.5	A	17.3	A	-	-		2.7	A	24.3	A	-	-		1437913_at
Bcl2l1	94.6	A	92.9	A	-		77.1	A	121.9	A	-	-		72.3	A	43.9	A	-	-		1420887_a_at
Bcl2l1	28.0	A	23.6	A	-		29.4	A	62.1	A	-	-		22.6	A	37.5	A	-	-		1426050_at
Bcl2l1	2.8	A	4.9	A	-		3.6	A	2.3	A	-	-		2.5	A	3.5	A	-	-		1426191_a_at
Bcl2l1	276.9	P	274.0	P	1.01		299.8	P	343.1	P	0.93	NC		211.9	P	331.9	P	0.66	NC		1420888_at
Bcl2l11	40.6	A	56.3	A	-		58.6	A	52.9	A	-	-		44.5	A	50.1	A	-	-		1426334_a_at
Bcl2l11	35.0	A	41.1	A	-		32.5	A	69.6	A	-	-		18.8	A	59.4	A	-	-		1435449_at
Bcl2l11	61.1	A	78.2	A	-		46.2	A	93.5	A	-	-		21.6	A	41	A	-	-		1456006_at
Bcl2l11	159.1	P	178.5	M	0.89		123.3	M	122	A	-	-		83.1	P	122.1	A	0.57	NC		1456005_a_at
Bcl2l11	139.7	P	147.0	P	0.95		207.3	P	195	P	1.00	NC		114.6	P	155.8	P	0.62	NC		1435448_at
Bcl2l2	66.4	A	78.3	A	-		64.8	A	91.6	A	-	-		47	A	80.1	A	-	-		1430453_a_at
Bcl2l2	45.6	A	51.4	A	-		53.4	A	16.7	A	-	-		29.2	A	19.6	A	-	-		1430454_x_at
Bcl2l2	51.0	A	52.1	A	-		47.5	A	54.4	A	-	-		27.3	A	61.1	A	-	-		1451029_at
Bcl2l2	373.4	P	402.1	P	0.93		336.7	P	353.3	P	0.93	NC		233.5	P	415	P	0.62	NC		1423572_at
Bid	9.6	A	3.4	A	-		5.2	A	2.6	A	-	-		2.3	A	3.6	A	-	-		1417045_at
Bid	6.0	A	6.0	A	-		22.9	A	3.2	A	-	-		7.4	A	14.8	A	-	-		1448560_at
Birc2	174.3	P	165.0	P	1.06		180.9	P	145.1	P	1.41	I		108.5	P	95.1	P	0.81	NC		1418854_at
Birc3	3.0	A	8.9	A	-		7.2	A	6.7	A	-	-		3.4	A	6	A	-	-		1425223_at
Birc3	183.1	P	138.6	A	1.32		237.8	P	116.8	A	1.52	NC		124.2	A	127.1	A	-	-		1421392_a_at
Birc4	8.5	A	8.9	A	-		17.6	A	24.3	A	-	-		9.9	A	9.7	A	-	-		1426636_a_at
Birc4	9.3	A	17.4	A	-		7.1	A	12.1	A	-	-		3.7	A	18.4	A	-	-		1450231_a_at
Birc4	33.0	A	10.6	A	-		18.9	A	19.1	A	-	-		25.2	A	25.6	A	-	-		1450232_at
Birc4	36.2	P	36.7	P	0.99		53.3	P	57.9	P	0.81	NC		15.2	P	3.8	A	2.64	NC		1456088_at
Birc4	211.7	P	238.1	P	0.89		261.2	P	238	P	1.15	NC		136.3	P	168	P	0.81	NC		1437533_at
Birc4 /// LOC38	9.1	A	9.1	A	-		13.1	A	16.7	A	-	-		6.6	A	1.3	A	-	-		1421394_a_at
Birc5	273.9	P	285.8	P	0.96		196	A	245.7	A	-	-		69.1	A	14.2	A	-	-		1424278_a_at
Bnip3l	478.1	P	402.9	P	1.19		507.8	P	411.5	P	1.07	NC		412.4	P	479.8	P	1.00	I		1416922_a_at
Bnip3l	266.7	P	180.5	P	1.48		295.9	P	297.6	P	1.15	NC		259.9	P	311.8	P	0.87	I		1416923_a_at
Bnip3l	118.9	P	112.2	P	1.06		115.4	P	120.8	P	0.93	NC		137.9	P	143.9	P	0.87	NC		1448525_a_at
Bok	541.3	P	457.6	P	1.18		310.6	P	557.7	P	0.62	D		161.7	P	397.2	P	0.50	NC		1417040_a_at
Casp1	16.5	A	7.1	A	-		40.8	M	43.1	A	-	-		19.8	M	32.4	A	-	-		1449265_at
Casp11	41.1	A	21.4	A	-		60.8	A	6.7	A	-	-		27.9	A	12.5	A	-	-		1449591_at
Casp12	104.4	P	73.1	P	1.43		135.6	P	96.7	A	1.41	I		117.8	P	89.1	P	1.00	NC		1418981_at

Casp12	58.5	P	52.9	P	1.11	99.4	P	48.7	P	1.52	NC	32.2	P	63.4	P	0.57	NC		1449297_at
Casp2	158.6	P	181.0	P	0.88	168.5	P	148.2	P	0.93	NC	96.8	P	115.9	P	0.66	NC		1448165_at
Casp3	38.2	A	51.2	A	-	35.4	A	60.4	A	-	-	38.5	A	45.9	A	-	-		1426165_a_at
Casp3	114.2	A	66.0	A	-	159.3	A	144.5	A	-	-	80.5	A	141.8	A	-	-		1449839_at
Casp6	111.3	P	102.4	A	1.09	129.5	A	161.6	M	-	-	58.3	P	142.4	M	0.57	NC		1415995_at
Casp7	56.9	A	71.7	A	-	33.9	A	50.9	A	-	-	41.5	A	47.5	A	-	-		1426062_a_at
Casp7	10.9	A	16.8	A	-	11.7	A	17.1	A	-	-	9.2	A	17.6	A	-	-		1434099_at
Casp7	4.0	A	3.7	A	-	3.4	A	54.4	A	-	-	12.8	A	16.9	A	-	-		1434100_x_at
Casp7	62.5	P	87.5	P	0.71	96.2	P	94.4	A	0.87	NC	60.7	A	74.7	A	-	-		1448659_at
Casp8	162.8	P	148.8	P	1.09	217.1	P	162.7	P	1.23	I	135	P	182.2	P	0.81	NC		1424552_at
Casp9	16.5	A	28.2	A	-	47.1	A	52.6	A	-	-	52.9	A	10.2	A	-	-		1426125_a_at
Casp9	61.9	P	39.0	P	1.59	27.9	A	40.4	A	-	-	35.3	A	65.5	A	-	-		1437537_at
Cflar	56.5	A	40.4	A	-	29.7	A	32	A	-	-	65.3	A	35.8	A	-	-		1449317_at
Cflar	58.8	P	50.5	A	1.16	58.6	A	46	A	-	-	12.6	A	58.8	A	-	-		1425686_at
Cflar	28.3	A	35.1	P	0.81	29.3	A	27.7	P	0.81	NC	9.9	A	18.4	A	-	-		1425687_at
Cflar	154.6	P	145.3	P	1.06	169.4	P	160.5	P	1.07	NC	117.2	P	155.8	P	0.71	NC		1424996_at
Cradd	70.4	A	79.1	P	0.89	79.3	P	105.9	A	0.87	NC	57.1	P	221.3	P	0.54	NC		1427505_a_at
Cycs	745.3	P	726.5	P	1.03	630.8	P	816.4	P	0.81	NC	322.2	P	428.5	P	0.81	I		1422483_a_at
Cycs	338.6	P	284.5	P	1.19	264.1	P	316.4	P	0.93	NC	181.2	P	276.7	P	0.71	NC		1422484_at
Cycs	1860.2	P	1490.5	P	1.25	1390.4	P	1674.9	P	0.81	NC	718.7	P	1017.7	P	0.71	NC		1456071_a_at
Cyct	17.8	P	25.6	P	0.69	26.5	A	25	A	-	-	15.8	P	37.3	P	0.29	NC		1450752_at
Daxx	310.0	P	307.7	P	1.01	289.1	P	263.2	P	1.15	NC	176.6	P	411.9	P	0.47	NC		1419026_at
Dffa	97.1	P	145.5	P	0.67	117.4	M	115.4	P	1.32	NC	69.9	M	128.3	P	0.57	NC		1450885_at
Dffb	42.4	A	48.4	A	-	48.3	A	44.9	A	-	-	38	A	54.9	A	-	-		1421229_at
Dffb	20.9	P	22.1	A	0.94	19.7	P	10.9	A	1.62	NC	9.3	A	14.2	A	-	-		1437051_at
Fadd	163.0	P	206.1	P	0.79	196.5	P	186.6	P	1.07	NC	117.5	P	208.1	P	0.66	NC		1416888_at
Fas	4.6	A	4.0	A	-	9.2	A	4.2	A	-	-	2.2	A	3.9	A	-	-		1460251_at
Fasl	23.2	A	28.7	A	-	41.4	A	47.2	A	-	-	10.5	A	11.7	A	-	-		1418803_a_at
Fasl	26.1	A	45.6	A	-	49.4	A	45.7	A	-	-	25.3	A	38.8	A	-	-		1449235_at
Hrk	112.6	P	157.5	P	0.71	182.8	P	139.1	M	1.15	NC	94.7	A	185.1	P	0.54	NC		1450366_at
Mcl1	606.5	P	549.9	P	1.10	603.7	P	627.5	P	0.93	NC	277.9	P	576	P	0.44	D	*	1416880_at
Mcl1	359.2	P	251.3	P	1.43	306.6	P	218.2	P	1.15	NC	264	P	340	P	0.50	NC		1416881_at
Mcl1	838.9	P	720.3	P	1.16	907.3	P	913.8	P	1.00	NC	434.3	P	693.7	P	0.50	NC		1437527_x_at
Mcl1	614.8	P	522.7	P	1.18	537.4	P	531.6	P	1.00	NC	328.4	P	552.7	P	0.62	NC		1448503_at
Mcl1	681.2	P	544.9	P	1.25	749.9	P	686.8	P	1.07	NC	281.9	P	683.7	P	0.38	D	*	1456243_x_at
Mcl1	268.7	P	246.0	P	1.09	358.8	P	371.6	P	1.00	NC	142.9	P	319.4	P	0.47	D	*	1456381_x_at
Parp1	359.2	P	336.6	P	1.07	182.6	A	280.4	A	-	-	134.8	A	173.6	A	-	-		1422502_at
Parp1	274.3	P	206.8	P	1.33	300.4	P	246.8	P	0.87	NC	104.3	A	146.6	M	-	-		1422503_s_at
Parp1	138.1	P	136.2	P	1.01	96.7	P	160.2	P	0.81	NC	62	P	92.9	P	0.54	NC		1435368_a_at
Pdcd1	7.2	A	8.1	A	-	9.2	A	10.7	A	-	-	8.1	A	7.3	A	-	-		1449835_at
Pdcd10	52.5	A	31.8	A	-	52.8	A	41.6	A	-	-	44.2	A	25	A	-	-		1448528_at
Pdcd10	220.6	P	204.2	P	1.08	147.6	P	153.9	P	1.23	NC	116.9	P	158	P	0.76	NC		1448527_at
Pdcd2	111.6	P	105.1	P	1.06	102.2	P	117.1	P	0.76	NC	81.1	P	96.4	P	0.81	NC		1423534_at
Pdcd5	338.8	P	297.9	P	1.14	257.7	M	314.6	P	0.93	NC	222.9	P	293	A	0.76	NC		1450638_at
Pdcd6	398.2	P	351.2	P	1.13	295.2	P	339.7	P	1.00	NC	338.5	P	410.6	P	0.76	NC		1416027_at
Pdcd6ip	45.1	A	32.6	A	-	38.8	A	38	A	-	-	26.9	P	63	A	0.57	NC		1460263_at
Pdcd6ip	179.9	P	203.7	P	0.88	228	P	173	M	1.00	NC	114.7	P	169.4	P	0.57	NC		1449674_s_at
Pdcd6ip	78.9	P	63.5	P	1.24	78.7	P	102.7	P	1.07	NC	24.5	P	68.3	P	0.44	NC		1415937_s_at
Pdcd6ip	96.6	P	104.9	P	0.92	104.6	P	141.4	P	0.81	NC	32.1	P	103.8	P	0.54	NC		1426184_a_at
Pdcd6ip	172.9	P	208.1	P	0.83	155.1	P	226.5	P	1.15	NC	140.3	P	178.2	P	0.66	NC		1448155_at
Pdcd7	47.5	A	37.5	A	-	47	A	54.1	A	-	-	42.4	A	66.3	A	-	-		1416377_at
Pdcd8	168.7	P	182.3	P	0.93	86.8	P	122.4	P	0.76	NC	96.1	P	180.9	P	0.62	NC		1418127_a_at
Perp	320.2	P	262.2	P	1.22	362	P	270.9	P	0.81	NC	281.3	P	384.3	P	0.81	I		1416271_at
Pycard	16.9	A	16.3	A	-	27.3	A	34.6	A	-	-	18.7	P	13.6	A	0.93	NC		1417347_at

Pycard	197.2	P	119.1	P	1.66	198.8	P	222.1	P	0.87	NC	230.7	P	243	P	1.00	NC	1417346_at
Traf1	4.0	A	4.3	A	-	3.3	A	15.6	A	-	-	1.4	A	24.3	A	-	-	1423602_at
Traf2	61.6	A	86.8	A	-	74.4	A	89.8	A	-	-	45.1	A	53.7	A	-	-	1451233_at
Traf3	12.4	A	28.4	A	-	15.6	A	46	A	-	-	24.9	A	32.8	A	-	-	1449149_at
Traf3	131.8	P	156.3	P	0.84	172.2	P	179.7	P	1.00	NC	93.8	P	113.7	P	0.81	NC	1418587_at
